# Association of the Triglyceride–Glucose Index With Gallstone Disease: A Systematic Review and Meta‐Analysis of Observational Studies

**DOI:** 10.1002/jgh3.70380

**Published:** 2026-03-06

**Authors:** Nirupma Gupta, Sushma Narsing Katkuri, Rekha Arcot, Sachin Kumar, Sushma Verma, Sorabh Lakhanpal, Sanjay Kumar, Jeffrin Reneus Paul, Prakasini Satapathy, Edward Mawejje

**Affiliations:** ^1^ Department of Anatomy, School of Medical Sciences and Research Sharda University Greater Noida India; ^2^ Department of Community Medicine Malla Reddy Instiute of Medical Sciences Hyderabad Telangana India; ^3^ Department of Surgery, Dr. D. Y. Patil Medical College, Hospital and Research Centre Dr. D. Y. Patil Vidyapeeth (Deemed‐To‐Be‐University), Pimpri Pune Maharashtra India; ^4^ Faculty of Pharmaceutical Sciences Graphic Era Hill University Dehradun India; ^5^ Centre for Promotion of Research Graphic Era Deemed University Dehradun India; ^6^ Department of Pharmaceutics Noida Institute of Engineering and Technology (Pharmacy Institute) Greater Noida India; ^7^ School of Pharmaceutical Sciences Lovely Professional University Phagwara India; ^8^ Centre for Research Impact & Outcome, Chitkara College of Pharmacy Chitkara University Rajpura Punjab India; ^9^ Saveetha Medical College and Hospital, Saveetha Institute of Medical and Technical Sciences Saveetha University Chennai India; ^10^ University Center for Research and Development Chandigarh University Mohali Punjab India; ^11^ School of Public Health Makerere University College of Health Sciences Kampala Uganda

**Keywords:** gallstone disease, insulin resistance, metabolic dysfunction, triglyceride–glucose index

## Abstract

**Background/Objective:**

Gallstone disease (GSD) is a common hepatobiliary condition closely associated with metabolic disturbances. The triglyceride–glucose index (TyG index), a widely used surrogate marker of insulin resistance, has gained attention as a potential indicator of gallstone risk. This systematic review and meta‐analysis aimed to synthesize observational evidence evaluating the association between TyG index and GSD in adults.

**Methods:**

Web of Science, Embase, and PubMed were searched from inception to December 2025 for relevant observational studies. Study quality was assessed using the Newcastle–Ottawa Scale (NOS). Adjusted odds ratios (aORs), standardized mean differences (SMDs), adjusted hazard ratios (aHRs), and prevalence estimates were pooled using a random‐effects model (REM). Sensitivity analyses, subgroup analyses, and assessment of publication bias were conducted.

**Results:**

Nine studies including more than 430 000 participants were included. For each one‐unit increase in the TyG index, the pooled odds ratio for GSD was 1.332 (95% CI: 1.213–1.462; *I* ^2^ = 0%). When comparing the highest versus lowest TyG categories, the summary OR was 1.678 (95% CI: 1.150–2.448). Participants with GSD had higher TyG values than those without GSD (pooled SMD: 0.29; 95% CI: 0.26–0.32). The pooled prevalence of GSD was 10.51% (95% CI: 5.40–19.44), with heterogeneity (*I* ^2^ = 99.8%). Subgroup analyses were performed according to body mass index (BMI), sex, diabetes, and race.

**Conclusion:**

Higher TyG index values are consistently linked to elevated risk of GSD. Given that TyG is inexpensive and routinely available, it may be useful for identifying individuals at a heightened risk of GSD. More longitudinal studies are needed to determine causation and to assess whether TyG‐based screening can improve GSD risk prediction or prevention.

## 1 Introduction

Gallstone disease (GSD) is a major global hepatobiliary disorder, affecting approximately 10%–20% of adults worldwide and up to 25%–30% of older populations [1]. In the United States alone, gallstones account for more than 20–25 million cholecystectomies annually and over $6 billion in healthcare expenditures, focusing on both the clinical and economic burden of this condition [2, 3]. Although obesity, female sex, dyslipidemia, and advancing age are established risk factors, emerging evidence highlights the broader influence of metabolic dysfunction in the pathophysiology of gallstones. Cholesterol supersaturation of bile—driven by hepatic metabolic alterations—remains the principal mechanism underlying most cholesterol stones, reinforcing the link between metabolic health and gallstone formation [4].

Insulin resistance (IR) represents a central metabolic abnormality unifying obesity, nonalcoholic fatty liver disease (NAFLD), type 2 diabetes mellitus (T2DM), and disturbances in hepatic lipid handling [5]. Mechanistic pathways through which IR contributes to gallstone formation include increased hepatic cholesterol synthesis, diminished bile acid production, altered biliary lipid secretion, and impaired gallbladder motility [6]. These metabolic alterations create a physiological environment that supports nucleation and growth of gallstones. Although conventional measures of IR, such as HOMA‐IR, provide useful insights, their reliance on insulin assays limits utility in many clinical and research settings, prompting interest in simpler, reproducible alternatives.

The triglyceride–glucose index (TyG index), calculated from FTG and glucose concentration, has gained recognition as a simple and reproducible surrogate of IR. It correlates well with the hyperinsulinemic–euglycemic clamp and has been shown to predict metabolic syndrome, NAFLD, cardiovascular disease, and several chronic inflammatory conditions [7]. Given this broad metabolic relevance, it is biologically plausible that TyG may also reflect risk pathways involved in gallstone development. Recent observational studies have begun to explore this relationship; however, findings vary considerably across populations, analytical methods, and outcome definitions, leaving uncertainty about the magnitude and consistency of the association.

Despite increasing interest in TyG as an integrative metabolic risk indicator, no comprehensive synthesis has evaluated its relationship with GSD across diverse study settings. Critical knowledge gaps remain, including whether TyG predicts gallstone risk independent of age, sex, body mass index (BMI), and racial or ethnic differences, and whether categorical high TyG levels or per‐unit increases yield stronger associations. Furthermore, the extent to which study design, diagnostic modality, and adjustment strategies influence the observed relationship is unknown. Therefore, this review was conducted to quantify the relationship between the TyG index and GSD, explore subgroup, assess the robustness, sensitivity analyses, and consistency of existing observational data.

## 2 Methods

This systematic review and meta‐analysis (SR‐MA) was planned and conducted following the guidelines provided in the Preferred Reporting Items for Systematic Reviews and Meta‐Analyses (PRISMA) checklist (Table S1). The analytical methods and inclusion criteria were pre‐established and thoroughly documented in the protocol registered on PROSPERO **(CRD420251250081)**.

### 2.1 Eligibility

The review included observational studies evaluating the association between GSD and the TyG index in adults (≥ 18 years). Eligible studies included human participants from community‐based or hospital‐based settings with clearly documented gallstone status confirmed through ultrasonography, CT/MRI, medical records, or validated self‐report measures. Cohort, case–control, and cross‐sectional designs (prospective or retrospective) were all eligible if the TyG index was reported or could be calculated from fasting triglyceride and glucose values. Both categorical classifications of TyG (e.g., tertiles, quartiles, quintiles) and continuous TyG values were eligible, with comparisons typically using the lowest TyG category or lower TyG values as the reference. Outcomes were required to provide effect estimates odds ratio (OR), risk ratio (RR), or hazard ratio (HR) or raw data suitable for computation.

Studies were excluded if they involved children or adolescents (< 18 years), pregnant‐only cohorts, animal or in vitro models, or genetic‐only analyses without TyG measurement. Studies reporting only TyG‐derived indices, such as the triglyceride–glucose–waist circumference index (TyG–WC), triglyceride–glucose–body mass index (TyG–BMI), or triglyceride–glucose–waist‐to‐height ratio (TyG–WHtR), without reporting the TyG index itself, were excluded. Additional exclusions applied to studies lacking gallstone‐related outcomes, those assessing biliary conditions unrelated to gallstones (such as isolated common bile duct stones or biliary sludge), cholecystectomies performed for non‐gallstone indications, and studies without extractable effect sizes. Reviews, case series, case reports, meta‐analyses, conference abstracts without full data, editorials, and interventional trials without observational baseline TyG–gallstone associations were also excluded. Only full‐text articles published in English up to December 2025 were considered (Table S2).

### 2.2 Search Strategy

Three databases—Web of Science, PubMed, and Embase—were systematically queried from their inception to December 2025. Search terms combined controlled vocabulary specific to each database (e.g., MeSH, Emtree) with keywords related to both the TyG index and GSD. Search strings incorporated terms such as *“triglyceride–glucose index,” “TyG,”* “insulin resistance surrogate,” and *“gallstone,” “cholelithiasis,” “biliary stones,”* and related biliary disorders. Boolean operators were applied to broaden or narrow retrieval as needed. Database‐specific adaptations ensured optimal capture of relevant records. No limits were placed on study design during the initial search stage (Table S3).

### 2.3 Screening and Data Extraction

The Nested Knowledge platform was used to oversee the research selection procedure. Potentially pertinent papers underwent full‐text review after titles and abstracts were checked by two independent reviewers. Any disagreements were settled by dialogue or the participation of a third reviewer.

Study‐level data were gathered using a standardized extraction form. Publication year, sample size, country, study setting, participant characteristics, gallstone diagnostic criteria, TyG calculation or stratification method, adjusted effect estimates with confidence interval (CI), and factors used in statistical adjustments were among the items collected.

### 2.4 Quality Assessment

The methodological quality of the included studies was appraised using the Newcastle–Ottawa Scale (NOS), with separate scoring approaches for different study designs. Two reviewers rated each study independently, and inconsistencies were resolved jointly. Cross‐sectional studies were evaluated using a modified six‐point NOS that assessed representativeness, sample adequacy, clarity of exposure and outcome definitions, and appropriateness of statistical analyses. For cohort and case–control designs, the original nine‐point NOS was applied, focusing on how participants were selected, how well the groups were matched, and how reliably exposures and outcomes were measured. Scores of 7–9 were considered high quality, 4–6 moderate quality, and ≤ 3 low quality [8].

### 2.5 Evidence Synthesis

All analyses were conducted using R (version 4.4) [9]. The primary measures of association extracted from the included studies were adjusted odds ratio (aOR) and adjusted hazard ratio (aHR) with corresponding 95% CI. Studies reporting OR and HR were synthesized separately to ensure consistency of effect measures. A random‐effects model (REM) meta‐analysis was used to compute pooled estimates that took into consideration both within‐study and between‐study variability. Separate meta‐analyses were performed for high versus low TyG index categories, per‐unit increases in TyG index, and TyG quantiles (tertiles or quartiles), where applicable.

Variability across studies was quantified using the *I* ^2^ statistic, with values below 25% indicating minimal heterogeneity, around 50% reflecting moderate levels, and 75% or higher signifying substantial heterogeneity. A *Q*‐test *p* < 0.10 was considered significant. To evaluate the influence of individual studies, leave‐one‐out sensitivity analyses were performed. Subgroup analyses were carried out when appropriate, considering variables such as age, BMI, diabetes status, race/ethnicity, and sex [10].

Potential publication bias was examined by constructing Doi plots and calculating the LFK index, with values beyond ±2 interpreted as indicative of major asymmetry. Statistical significance for all analyses was set at *p* < 0.05, unless stated otherwise [11, 12].

## 3 Results

### 3.1 Study Selection

Across the three electronic databases, 78 records were initially retrieved: 22 from Web of Science, 31 from Embase, and 25 from PubMed. After removing 43 duplicates, 35 unique records proceeded to title and abstract screening. Twenty‐three were excluded for clear irrelevance, leaving 12 articles for full‐text examination. Of these, five did not meet the inclusion criteria—two were reviews, two lacked an appropriate study population, and one did not report relevant outcomes.

**FIGURE 1:**
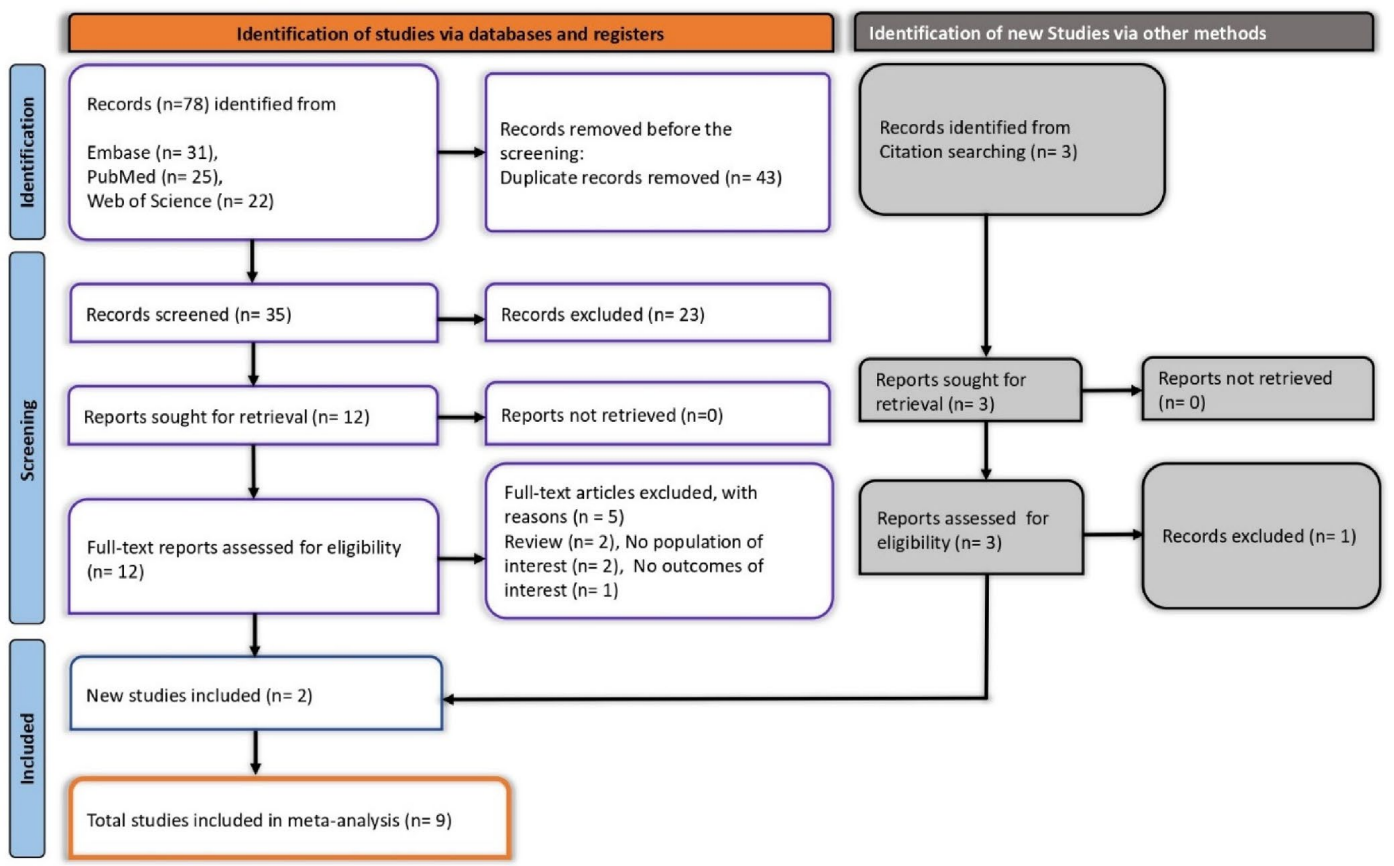
PRISMA flow chart showing the studies selection process.

An additional three articles were located through citation screening, and two of these satisfied the eligibility criteria. Ultimately, nine studies qualified for inclusion in the final synthesis (Figure 1).

### 3.2 Quality Assessment of Included Studies

Overall, the methodological quality of the included literature was satisfactory. Among the five cross‐sectional studies assessed with the modified NOS, four were rated as high quality (scores of 5–6), while one was rated moderate quality (score of 4).

All four cohort or case–control studies evaluated with the original NOS scored between 7 and 9, classifying them as high methodological quality (Tables S4 and S5).

### 3.3 Summary Characteristics of Included Studies

The nine included observational studies comprised one case–control study, two cohort studies, and six cross‐sectional analyses, conducted between 2006 and 2022. The National Health and Nutrition Examination Survey (NHANES) in the United States provided data for six [13, 14, 15, 16, 17, 18] of the investigations; the remaining studies were carried out in China (*n* = 1) [19], the UK (*n* = 1) [20], and Turkey (*n* = 1) [21].

Across all studies, the combined sample exceeded 430 000 participants, individual study sizes ranging from 210 to 395 391 participants. The number of participants with GSD ranged from 105 to 12 770, whereas the number of participants without GSD ranged from 105 to 395 391 across studies. The proportion of male participants ranged from approximately 24.7% to 52.5%. The mean or median age of study populations ranged from approximately 47 to 60 years. Several studies reported age separately for participants with and without GSD.

The TyG index values were reported either as mean with SD or as median. Among studies reporting group‐wise TyG values, the TyG index ranged from approximately 8.65 to 9.04 in GSD groups and from 8.43 to 8.64 in non‐GSD groups. In the case–control study from Turkey, the TyG index was reported as 9.04 in the gallstone group and 8.71 in the control group. Cohort studies from the UK and China reported TyG exposure at baseline with gallstone outcomes assessed during follow‐up (Table 1).

**TABLE 1:** Summary characteristics of included studies.

Study	Study design	Study duration	Study data	Male %	Country	Mean age	TyG index	GSD	Without GSD	Total
Feng 2024 [13]	Cross‐sectional study	2017 to 2020	NHANES	GSD: 28.53 without GSD: 50.78	United States	GSD: 58.41 without GSD: 49.96	GSD: 8.82 without GSD: 8.64	838	6956	7794
Gong 2024 [14]	Cross‐sectional study	2017–2020	NHANES	GSD: 3.046 Without GSD: 47.158	United States	50.44	**Median GSD: 8.784 Without GSD: 8.572	602	5045	5647
Gong 2025 [15]	Cross‐sectional study	2017 to 2020	NHANES	Without GSD: 52.47 GSD: 23.20	United States	47	Without GSD: 8.43 GSD: 8.65	305	2506	2811
Li 2024 [16]	Cross‐sectional study	2017 to 2020	NHANES	Without GSD: 50.76 GSD: 28.78	United states	Without GSD: 49.98 GSD: 57.57	Without GSD: 8.49 GSD: 8.71	403	3467	3870
Li 2025 [20]	Cohort study	2006 to 2010	NA	45.46	United Kingdom	58 (Median)	NA	12 770	NA	395 391
Öztürk 2024 [21]	Case–control study	Jan 2020 to June 2022	NA	24.74	Turkey	52	GSD: 9.04 Control: 8.71	105	105	210
Tu 2025 [17]	Cross‐sectional study	2017 to 2020	NHANES	Without GSD = 33.54 GSD = 50.24	United States	Without GSD: 50.99 GSD: 50.70	Without GSD: 8.64 ± 0.65 GSD: 8.82 ± 0.62	836	6970	7806
Wang 2024 [18]	Cohort study	2017 to 2020	NHANES	Without GSD: 50.7 GSD: 28.0	United States	Without GSD: 51.00 GSD: 60.00	Without GSD: 8.60 GSD: 8.83	421	3484	3905
Zhang 2025 [19]	Cohort study	2016 to 2021	Questionnaire survey	GSD: 30.9 Without GSD: 52.2	China	GSD: 49.89 Without GSD: 37.13	GSD: 9.02 Without GSD: 8.60	466	10 354	10 820

### 3.4 Summary of Effect Sizes and Adjusted Confounders

All included studies reported multivariable‐adjusted associations between the TyG index and GSD. Eight studies reported aOR, while one cohort study reported aHR. The variables included for adjustment differed across studies but consistently involved major demographic, metabolic, and lifestyle factors, including age, race or ethnicity, sex, BMI, alcohol consumption, smoking status, education, diabetes, income level, physical activity, HTN, lipid parameters, renal function indices, and cardiovascular comorbidities.

Six studies reported results for high versus low TyG categories. The adjusted effect estimates for this comparison ranged from OR 1.36 (95% CI, 1.15–1.61) to OR 2.28 (95% CI, 1.83–2.86). Three studies additionally evaluated the association using per‐unit increases in TyG, with adjusted effect estimates ranging from OR 1.25 (95% CI, 1.04–1.51) to OR 1.51 (95% CI, 1.33–1.71). One cohort study reported a per‐unit TyG‐associated HR of 1.28 (95% CI, 1.10–1.49).

Five studies assessed TyG using quantile‐based categorizations including tertiles and quartiles. In these analyses, the lowest TyG category served as the reference group. The reported risk estimates increased across higher TyG categories, with the highest categories showing effect estimates ranging between OR 1.40 and OR 2.48. One cohort study similarly demonstrated increasing hazard ratios across TyG quartiles.

Several studies reported subgroup results stratified by age, BMI, diabetes status, sex, hypertension (HTN), smoking, race or ethnicity, and alcohol consumption (Table 2).

**TABLE 2:** Summary characteristics of effect size and its adjusted confounder.

Study	Effect size	Confounder	High vs low TyG	Per‐unit TyG	TyG quartile
Feng 2024	OR (95% CI) Stratified by gender—Male: 1.26 (1.00, 1.57) Female: 1.33 (1.11, 1.59) Stratified by race—Mexican American: 0.99 (0.66, 1.50) White people: 1.25 (0.82, 1.91) Black people: 1.36 (1.13, 1.63) Other Race: 1.43 (0.99, 2.05) Stratified by age (years)—20–39: 1.62 (1.12, 2.33) 40–59: 1.24 (0.98, 1.58) 60–85: 1.22 (1.00, 1.49)	Gender, age, race, educational attainment, poverty‐to‐income ratio (PIR), alcohol consumption, cholesterol levels, uric acid, smoking habits, creatinine levels, history of asthma, HTN, diabetes, coronary heart disease, and cancer.	OR = 1.36 (95% CI: 1.15, 1.61)	OR = 1.28 (95% CI: 1.12, 1.47)	NA
Gong 2024	OR (95% CI) BMI—< 25: 1.06 (0.81–1.38) 25–29: 1.04 (0.89–1.23) ≥ 30: 1.14 (1.01–1.28) Age—18–39: 1.25 (1.02–1.54) 40–59: 1.13 (0.97–1.33) ≥ 60: 1.01 (0.89–1.36) Gender—Female: 1.15 (1.03–1.28) Male: 1.04 (0.88–1.22) Married—Others: 1.17 (1.01–1.35) Yes: 1.06 (0.94–1.19) Diabetes—No: 1.13 (1.02–1.25) Yes: 1.00 (0.82–1.21) Smoking—No: 1.20 (1.05–1.37) Yes: 1.01 (0.89–1.35) Alcohol—No: 1.01 (0.85–1.20) Yes: 1.14 (1.03–1.27)	Gender, age, race, PIR, BMI, education, marital status, smoking, alcohol use, diabetes, HTN	NA	NA	Q1, < 8.19: Ref Q2, ≥ 8.19, < 8.59: 1.33 (0.99, 1.78) Q3, ≥ 8.59, < 9.03: 1.51 (1.13, 2.02) Q4, ≥ 9.03 2.48: 1.40 (1.04, 1.89)
Gong 2025	OR (95% CI) Gender— Female: 2.30 (1.60–3.30) Male: 1.59 (0.95–2.67)	Age, sex, race, marital status, education attainment, income level, smoking history, alcohol consumption, physical activity, and the comorbidity of diabetes and HTN.	NA	1.197 (0.798, 1.794)	Q4 versus Q1: 1.596 (0.723, 3.524)
Li 2024	OR (95% CI) Gender—Male: 1.37 (0.87, 2.17) Female: 1.70 (1.17, 2.48) Alcohol—Yes: 1.67 (1.15, 2.43) No: 1.30 (0.77, 2.19) BMI—< 25: 1.28 (0.97, 1.69) > = 25, < 29.9: 1.29 (1.06, 1.56) > = 29.9: 1.81 (1.19, 2.76) Smoke status—Yes: 1.68 (1.09, 2.57) No: 1.62 (1.10, 2.38) Diabetes—Yes: 1.31 (0.80, 2.17) No: 1.67 (1.16, 2.41) Hypertension—Yes: 1.58 (1.04, 2.41) No: 1.67 (1.13, 2.47) Total cholesterol—< 200 mg/dL: 1.51 (0.96, 2.37) > 200 mg/dL: 1.63 (1.13, 2.36)	Gender, age, race, education level, marital status, PIR, alcohol consumption, smoking status, HTN, diabetes, coronary heart disease, BMI, fasting glucose level, low‐density cholesterol level, triglyceride level, total cholesterol level, and waist circumference (WC).	(OR 1.93, 95% CI 1.27, 2.94)	OR = 1.41, 95% CI 1.07, 1.86	NA
Li 2025	HR (95% CI) Gender— Female: 1.29 (1.31 to 1.38) Male: 1.24 (1.15 to 1.32) Age—38–44: 1.50 (1.29–1.74) 45–59: 1.26 (1.17–1.35) 60–73: 1.23 (1.15–1.32) BMI—< 18.5: 7.47 (2.80–19.95) 18.5–24.9: 1.71 (1.53–1.92) 25–29.9: 1.28 (1.19–1.38) > = 30: 1.12 (1.05–1.21)	NA	NA	HR (95% CI): 1.282 (1.100–1.494)	Q1 (< 8.31): Reference Q2 (8.31–8.67): 1.526 (1.253–1.859) Q3 (8.68–9.06): 1.537 (1.252–1.888) Q4 (≥ 9.07): 1.456 (1.137–1.864)
Öztürk 2024	Mean (SD) BMI Groups Normal weight 9.03 (0.58) Overweight 8.94 (0.38) Obese 9.11 (0.36)	NA	NA	NA	NA
Tu 2025	OR (95% CI) Gender—Male: 1.48 (1.23–1.76) Female: 1.54 (1.30–1.82) Stratified by age (yr)—< 60: 1.58 (1.35–1.85) ≥ 60: 1.40 (1.15–1.71) Stratified by race—Mexican American: 1.46 (1.01–2.12) Other Hispanic: 1.82 (1.28–2.58) Non‐Hispanic White: 1.43 (1.16–1.76) Non‐Hispanic Black: 1.28 (1.01–1.64) Other race: 1.94 (1.43–2.64) Diabetes—Yes: 1.52 (1.12–2.05) No: 1.52 (1.32–1.74) Hypertension—Yes: 1.40 (1.15–1.70) No: 1.59 (1.35–1.86)	Sex, age, race/ethnicity, education, PIR, BMI, weight, marital status (married/living with partner vs. single), alcohol consumption (current drinking vs. not), physical activity (vigorous, moderate, or less than moderate), smoking status, HTN, diabetes, asthma, cancer, CVD, and dietary intake (energy, fat, sugar, and water)	OR = 2.28 (95% CI: 1.83–2.86)	OR = 1.51 (95% CI: 1.33–1.71)	OR (95% CI) Tertile 1: 1.0 Tertile 2: 1.65 (1.31–2.07) Tertile 3: 2.28 (1.83–2.86)
Wang 2024	OR (95% CI) Stratified by age (years)—20–39: 2.02 (1.23–3.29) 40–59: 1.24 (0.91–1.68) 60–80: 1.09 (0.81–1.45) Stratified by gender—Female: 1.39 (1.09–1.77) Male: 1.12 (0.82–1.52) Stratified by race—White: 1.31 (0.96–1.81) Black: 1.02 (0.63–1.66) Mexican American: 0.85 (0.46–1.57) Other Race: 1.46 (1.06–2.02) Stratified by BMI—≤ 24.9: 1.24 (0.68–2.25) 25–29.9: 0.89 (0.60–1.32) ≥ 30: 1.07 (0.83–1.39) Stratified by diabetes—No: 1.37 (1.07–1.74) Yes: 1.05 (0.76–1.46)	Age, gender, race, BMI, diabetes, asthma, HTN, cancer, CHD, energy intake, fat intake, sugar intake, water intake, TG, FBG, SCr, education level, PIR, marital status, alcohol consumption, physical activity level, smoking status	NA	(OR = 1.25, 95% CI: 1.04, 1.51)	Tertile 1 (OR = 1.48, 95% CI: 1.09, 1.99)

### 3.5 Meta‐Analysis

#### 3.5.1 Impact of Per‐Unit Increase in TyG Index on the Risk of Gallstone Disease

**FIGURE 2:**
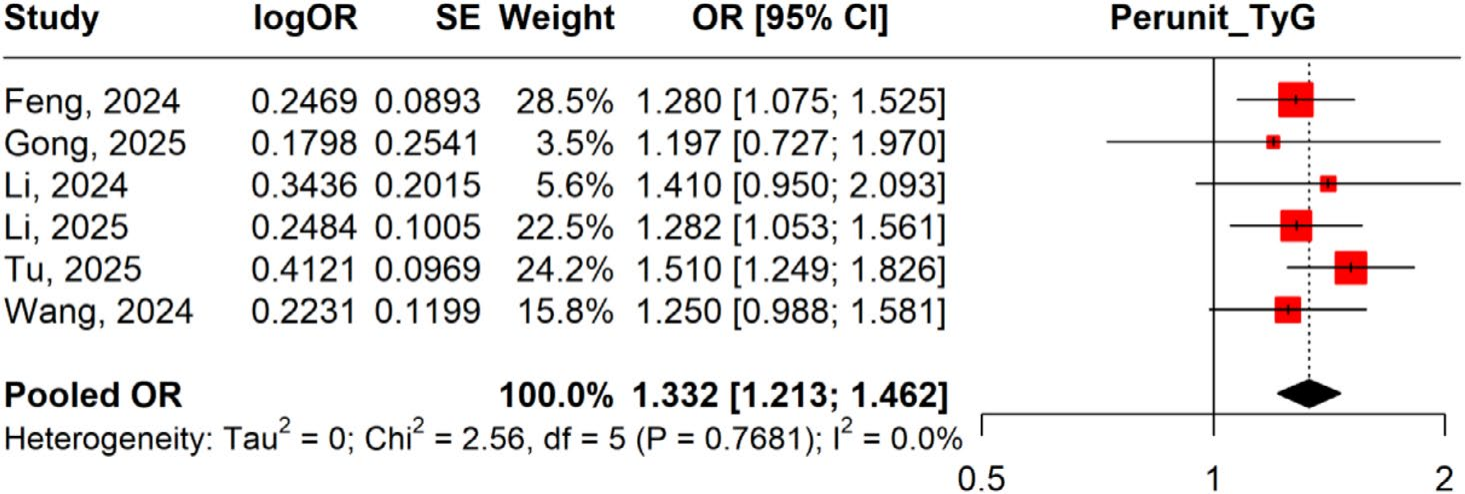
Forest plot illustrating the impact of per‐unit increase in TyG Index on the risk of gallstone disease.

From six studies, the pooled OR was 1.332 (95% CI: 1.213–1.462) with heterogeneity (*I* ^2^ = 0.0%) (Figure 2). Leave‐one‐out analyses demonstrated stable findings, with no single study disproportionately influencing the results (Figure S1).

#### 3.5.2 High Versus Low TyG Index on the Risk of Gallstone Disease

**FIGURE 3:**
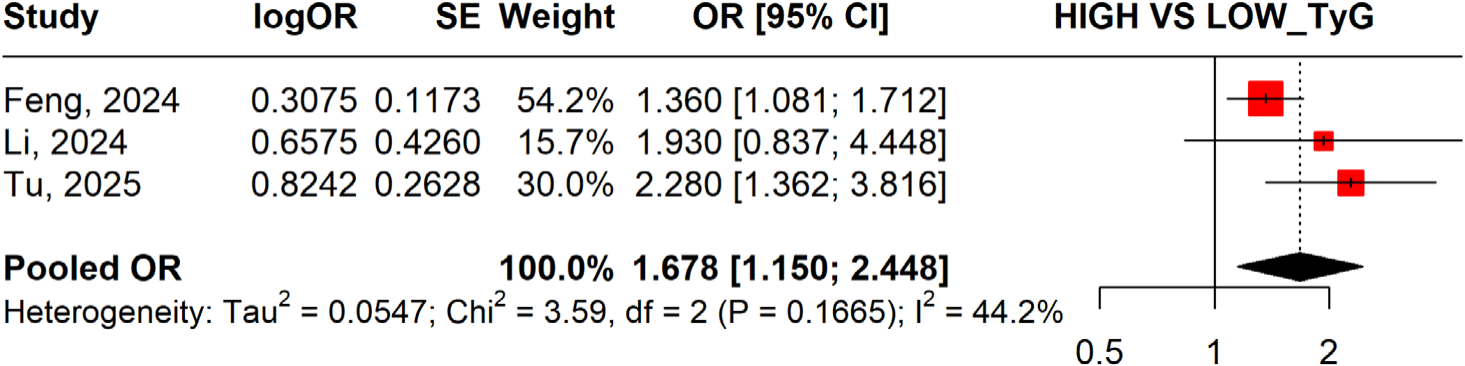
Forest plot illustrating the high versus low TyG Index on the risk of gallstone disease.

From three studies, the pooled OR was 1.678 (95% CI: 1.150–2.448) with heterogeneity (*I* ^2^ = 44.2%) (Figure 3). Sensitivity analysis showed similar overall results, although omitting the Li 2024 resulted in a pooled OR of 1.669 (95% CI: 1.017–2.739) with heterogeneity (*I* ^2^ = 69.0%) (Figure S2).

### 3.6 Subgroup Analysis

#### 3.6.1 Age

Subgroup analysis according to age categories demonstrated varying pooled effect sizes. Among participants aged 20–39 years, the pooled OR was 1.331 (95% CI: 1.055–1.680), with heterogeneity (*I* ^2^ = 0%). In the 40–59 years subgroup, the pooled OR was 1.234 (95% CI: 1.143–1.331), with heterogeneity (*I* ^2^ = 0%). For participants aged 60–85 years, the pooled OR was 1.185 (95% CI: 1.081–1.298), with heterogeneity (*I* ^2^ = 0%) (Table 3).

**TABLE 3:** Subgroup analysis based on age, BMI, diabetes status, gender and race.

Subgroup		Study no.	OR	Lower limit	Upper limit	Heterogeneity
Age						
	20–39	3	1.331	1.055	1.680	0%
	40–59	4	1.234	1.143	1.331	0%
	60–85	4	1.185	1.081	1.298	0%
BMI						
	≤ 25	3	1.147	0.925	1.422	8%
	25–29	4	1.155	0.996	1.339	59.7%
	≥ 30	4	1.126	1.053	1.203	0%
Diabetes status						
	Yes	4	1.083	0.915	1.281	0%
	No	4	1.326	1.095	1.607	59%
Gender						
	Female	7	1.292	1.190	1.403	27.5%
	Male	7	1.217	1.099	1.347	5.3%
Race						
	Black people	2	1.287	1.028	1.612	0%
	Mexican American	3	1.054	0.792	1.405	0%
	Other race	3	1.560	1.148	2.120	0%
	White people	2	1.287	1.028	1.612	0%

#### 3.6.2 Body Mass Index (BMI)

Subgroup analysis according to BMI categories demonstrated varying pooled effect sizes. Among participants with BMI < 25 kg/m^2^, the pooled OR was 1.147 (95% CI: 0.925–1.422), with heterogeneity (*I* ^2^ = 0%). In the BMI 25–29 kg/m^2^ subgroup, the pooled OR was 1.155 (95% CI: 0.996–1.339), with heterogeneity (*I* ^2^ = 59.7%). For participants with BMI ≥ 30 kg/m^2^, the pooled OR was 1.126 (95% CI: 1.053–1.203), with heterogeneity (*I* ^2^ = 0%) (Table 3).

#### 3.6.3 Diabetes Status

Subgroup analysis according to diabetes status demonstrated varying pooled effect sizes. Among participants with diabetes, the pooled OR was 1.083 (95% CI: 0.915–1.281), with heterogeneity (*I* ^2^ = 0%). In participants without diabetes, the pooled OR was 1.326 (95% CI: 1.095–1.607), with heterogeneity (*I* ^2^ = 59%) (Table 3).

#### 3.6.4 Gender

Gender‐based subgroup analysis demonstrated the following results. In the female subgroup, seven studies were included in the pooled analysis. The combined OR was 1.292 (95% CI: 1.190–1.403) with heterogeneity (*I* ^2^ = 27.5%) (Table 3). For the male subgroup, seven studies were included in the pooled analysis. The combined OR was 1.217 (95% CI: 1.099–1.347), with heterogeneity (*I* ^2^ = 5.3%) (Table 3).

#### 3.6.5 Race

Race‐based subgroup analysis demonstrated the following results. Among Mexican American participants, the pooled OR was 1.054 (95% CI: 0.792–1.405), with heterogeneity (*I* ^2^ = 0%). In the Other Race subgroup, the pooled OR was 1.560 (95% CI: 1.148–2.120), with heterogeneity (*I* ^2^ = 0%). Among White participants, the pooled OR was 1.287 (95% CI: 0.920–1.799), with heterogeneity (*I* ^2^ = 0%). For Black participants, the pooled OR was 1.287 (95% CI: 1.028–1.612), with heterogeneity (*I* ^2^ = 0%) (Table 3).

### 3.7 Difference in TyG Index Between Participants With and Without Gallstone Disease

**FIGURE 4:**
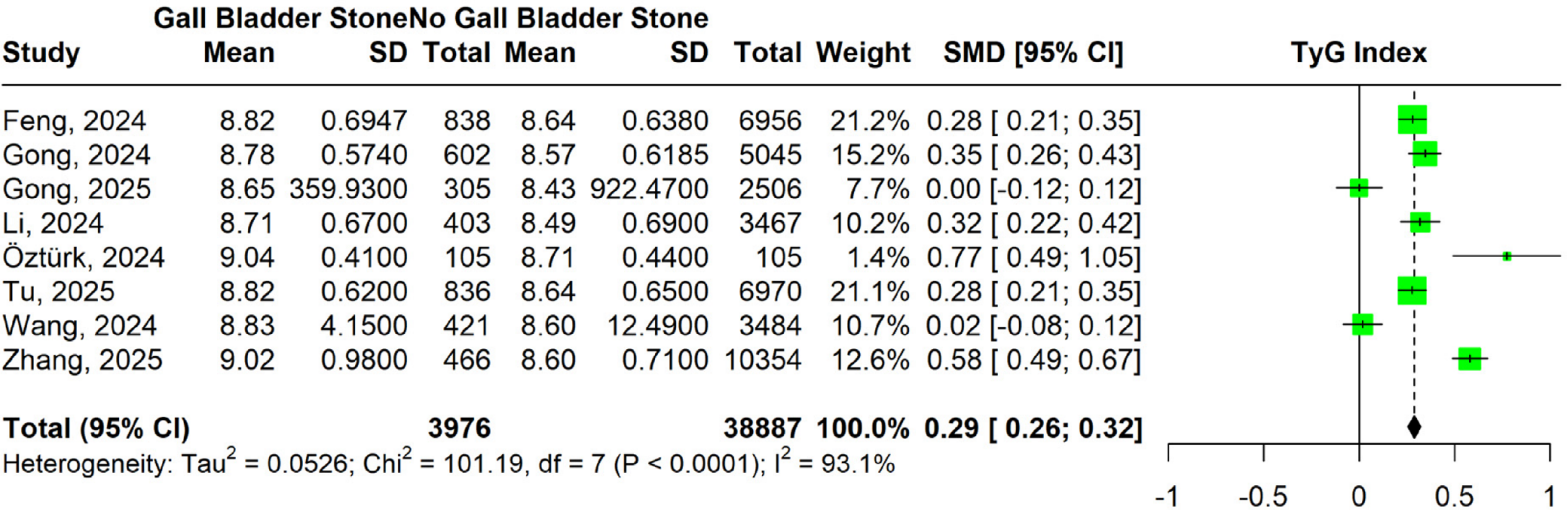
Forest plot illustrating the difference in TyG Index between participants with and without gallstone disease.

The meta‐analysis of eight studies compared TyG index levels between participants with GSD and those without GSD. The pooled SMD in TyG index was 0.29 (95% CI: 0.26–0.32) with heterogeneity (*I* ^2^ = 93.1) (Figure 4).

### 3.8 Prevalence of Gallstone Disease

**FIGURE 5:**
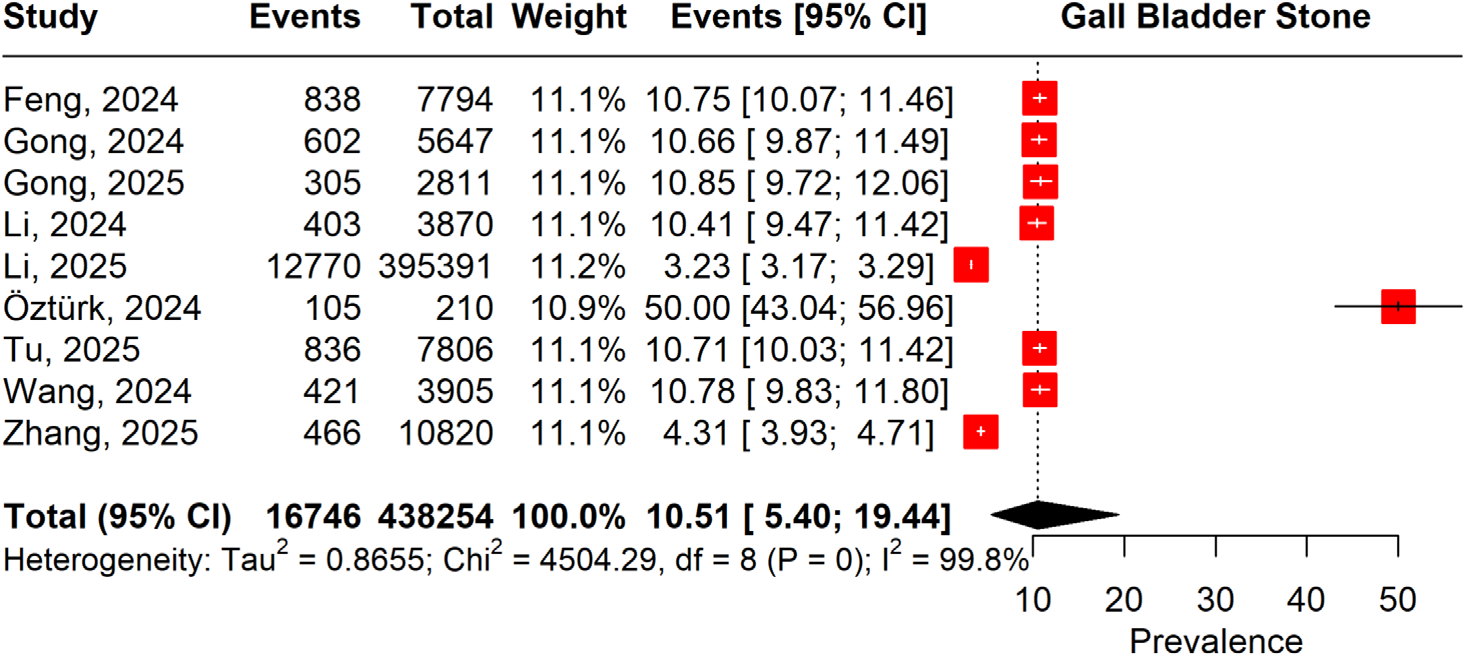
Forest plot illustrating the prevalence of gallstone disease.

Across the 9 studies, the overall pooled prevalence of GSD was 10.51% (95% CI: 5.40–19.44). Prevalence estimates varied widely (3.23% to 50%), and heterogeneity was extreme (*I* ^2^ = 99.8%) (Figure 5). Leave‐one‐out analyses demonstrated stable findings, with no single study disproportionately influencing the results (Figure S3).

### 3.9 Publication Bias

Publication bias assessment using the Doi plot showed pronounced asymmetry. The LFK index was 6.37, indicating major asymmetry and suggesting substantial publication bias (Figure S4).

## 4 Discussion

The TyG index and GSD are consistently and statistically significantly correlated, according to an SR‐MA of over 430 000 people. There was almost no between‐study heterogeneity and about a 33% increase in GSD risk for every unit rise in TyG. Additionally, participants with GSD had significantly greater TyG levels than those without, and those in the highest TyG categories had significantly higher odds of GSD than those in the lowest categories. These results validate the TyG index as a reliable metabolic indicator of gallstone risk.

Although IR has long been implicated in gallstone pathogenesis, previous evidence has focused on indirect metabolic constructs rather than simple IR surrogates. Earlier meta‐analyses demonstrated that metabolic syndrome and its components—including obesity, hypertension, dyslipidemia, and hyperglycemia—are independently associated with GSD, with risk increasing as the number of metabolic abnormalities accumulates [22]. Large‐scale lipid‐based studies further confirmed that elevated triglycerides and reduced HDLC are significant predictors of GSD [23]. More recently, metabolomic evidence has shown that bile acid dysregulation is a central feature of gallstone formation. The present meta‐analysis extends this literature by uniquely demonstrating that the TyG index—integrating both glucose and triglyceride metabolism into a single marker—independently predicts gallstone risk beyond traditional metabolic indicators [24].

Beyond GSD, the triglyceride–glucose index has been extensively evaluated as a surrogate marker of IR across a range of cardiometabolic conditions [25]. Previous systematic reviews and meta‐analyses have reported significant associations between elevated TyG index values and adverse outcomes in heart failure, obstructive sleep apnea, type 2 diabetes mellitus, peripheral artery disease, and atrial fibrillation. These findings support the broader relevance of the TyG index as an integrative metabolic marker and provide additional context for its observed association with GSD in the present analysis.

The biological plausibility of this association is supported by established mechanistic pathways linking IR to cholesterol gallstone formation. IR promotes hepatic cholesterol overproduction, reduces bile acid synthesis, and alters biliary lipid secretion, leading to cholesterol supersaturation of bile [26, 27]. Hypertriglyceridemia further increases hepatic lipid flux, while gallbladder hypomotility associated with metabolic dysfunction promotes bile stasis and crystal nucleation. Persistent low‐level inflammation and oxidative imbalance can further disrupt normal biliary physiology. Together, these interrelated processes help explain why elevated TyG values are linked to a greater likelihood of gallstone formation.

Subgroup analyses revealed important effect modification by demographic and metabolic characteristics. The association between TyG and GSD was strongest among younger adults and gradually weakened with advancing age, suggesting a more prominent etiologic role of metabolic dysfunction earlier in life. The relationship remained significant in both women and men, with slightly stronger effects observed in women, consistent with known sex‐related differences in gallstone prevalence [28]. The association was most evident among individuals with obesity but was attenuated among normal‐weight individuals, highlighting the synergistic interaction between IR and adiposity. The association was also stronger in participants without diabetes, possibly reflecting metabolic risk before pharmacologic modification.

From a clinical perspective, these findings may have potential implications for metabolic risk stratification in GSD. Because the TyG index is derived from routinely available laboratory measurements, it may help identify individuals with an increased metabolic susceptibility to gallstones, particularly younger adults and those with obesity [29]. This supports a preventive strategy focused on metabolic risk modification through weight control, dietary optimization, physical activity, and insulin‐sensitizing strategies, which may contribute to reducing future gallstone burden.

### 4.1 Strengths and Limitations

The analysis also has limitations. Considerable heterogeneity was observed for the pooled prevalence of GSD and for mean differences in TyG between GSD and non‐GSD groups, likely reflecting variations in diagnostic methods, population composition, diet, lifestyle, and genetic background. Significant publication bias was found, indicating that the underrepresentation of smaller studies with null results could inflate pooled values. Additionally, most of the included studies were cross‐sectional, making it impossible to draw clear conclusions about causality and timing. TyG was generally measured at a single point in time, and gallstone diagnosis varied across studies, raising the possibility of outcome misclassification. It is impossible to completely rule out residual confounding caused by unmeasured variables such as drug use, fast weight changes, pregnancy history, and specific food habits.

Despite these limitations, the study has several strengths, including a large aggregated sample size, an up‐to‐date and comprehensive search strategy, protocol registration, systematic ROB assessment using the NOS, and consistent associations across different TyG definitions and study designs. Future research should prioritize large prospective cohort studies with repeated TyG measurements and standardized gallstone assessment, as well as interventional studies that target IR, to determine whether lowering TyG levels can reduce the incidence of GSD.

## 5 Conclusion

Higher TyG index values are consistently associated with an increased risk of GSD. Because the TyG index relies on common laboratory tests and is simple and inexpensive to compute, it has potential utility as a practical tool for individuals who may be metabolically predisposed to gallstones. These results further emphasize the contribution of IR and broader metabolic disturbances to gallstone formation. Nonetheless, robust prospective studies are required to clarify causality and to assess whether integrating TyG into clinical risk evaluation can enhance early identification or preventive efforts. Improving metabolic health at the population level may ultimately help lessen the growing burden of GSD.

## Funding

The authors have nothing to report.

## Ethics Statement

The authors have nothing to report.

## Consent

The authors have nothing to report.

## Conflicts of Interest

The authors declare no conflicts of interest.

## Supporting information


**Table S1:** PRISMA checklist.
**Table S2:** Inclusion and exclusion criteria.
**Table S3:** The adjusted search terms as per searched electronic databases.
**Table S4:** Modified Newcastle–Ottawa Scale for the quality assessment of included studies.
**Table S5:** Newcastle–Ottawa Scale for the quality assessment of included studies.
**Figure S1:** Leave‐one‐out analysis representing results of impact of per‐unit increase in TyG Index on the risk of gallstone disease.
**Figure S2:** Leave‐one‐out analysis representing results of the high vs. low TyG Index on the risk of gallstone Disease.
**Figure S3:** Leave‐one‐out analysis representing results of prevalence of gallstone disease.
**Figure S4:** DOI plot depicting the publication bias of prevalence of gallstone disease.

## Data Availability

The data that supports the findings of this study are available in the Supporting Information of this article.
